# All-fibre supercontinuum laser for *in vivo* multispectral photoacoustic microscopy of lipids in the extended near-infrared region

**DOI:** 10.1016/j.pacs.2020.100163

**Published:** 2020-01-27

**Authors:** Manoj K. Dasa, Gianni Nteroli, Patrick Bowen, Giulia Messa, Yuyang Feng, Christian R. Petersen, Stella Koutsikou, Magalie Bondu, Peter M. Moselund, Adrian Podoleanu, Adrian Bradu, Christos Markos, Ole Bang

**Affiliations:** a DTU Fotonik, Technical University of Denmark, 2800 Kgs. Lyngby, Denmark; b NORBLIS IVS, Virumgade 35D, 2830 Virum, Denmark; c NKT Photonics A/S, Blokken 84, 3460 Birkerød, Denmark; d Applied Optics Group, University of Kent, Canterbury, UK; e Medway School of Pharmacy, University of Kent, Chatham, UK; f COPAC A/S, Diplomvej 381, 2800 Kongens Lyngby, Denmark

**Keywords:** Photoacoustic microscopy, Fibre lasers, Supercontinuum, Lipids

## Abstract

Among the numerous endogenous biological molecules, information on lipids is highly coveted for understanding both aspects of developmental biology and research in fatal chronic diseases. Due to the pronounced absorption features of lipids in the extended near-infrared region (1650−1850 nm), visualisation and identification of lipids become possible using multi-spectral photoacoustic (optoacoustic) microscopy. However, the spectroscopic studies in this spectral region require lasers that can produce high pulse energies over a broad spectral bandwidth to efficiently excite strong photoacoustic signals. The most well-known laser sources capable of satisfying the multi-spectral photoacoustic microscopy requirements (tunability and pulse energy) are tunable nanosecond optical parametric oscillators. However, these lasers have an inherently large footprint, thus preventing their use in compact microscopy systems. Besides, they exhibit low-repetition rates. Here, we demonstrate a compact all-fibre, high pulse energy supercontinuum laser that covers a spectral range from 1440 to 1870 nm with a 7 ns pulse duration and total energy of 18.3 μJ at a repetition rate of 100 kHz. Using the developed high-pulse energy source, we perform multi-spectral photoacoustic microscopy imaging of lipids, both *ex vivo* on adipose tissue and *in vivo* to study the development of *Xenopus laevis* tadpoles, using six different excitation bands over the first overtone transition of C–H vibration bonds (1650−1850 nm).

## 1 Introduction

The ability to visualise specific endogenous biological molecules following their spatial distribution and temporal dynamics *in vivo* without the use of perturbative labels is essential for understanding their physiological impact and subsequent regulatory mechanisms. To this end, photoacoustic microscopy (PAM) enables real-time visualisation of various endogenous agents, such as haemoglobin and melanin, using their inherent wavelength-dependent absorption at extended penetration depths. This label-free ability of PAM makes it a promising technique for the detection, diagnosis, and monitoring of various diseases [[1], [2], [3], [4], [5]].

Lipids play a crucial role in cellular physiology as structural components of biological membranes, biosynthetic precursors, and energy storage [6]. Moreover, they act as major contrast agents in the identification of fatal chronic diseases like atherosclerosis and myocardial infarction [14]. Therefore, there is a demand for high-resolution label-free imaging of lipids in medical imaging [[6], [7], [8], [9], [10], [11], [12], [13]]. However, early studies based on PAM revealed that the dominant absorption features of other endogenous agents, including haemoglobin and melanin, prevent effective imaging of lipids in the classical optical imaging window (400–700 nm) [14,15].

Following these early studies, attention turned to optical imaging windows in the longer near-infrared (NIR) wavelength regions: 1100−1300 nm and 1650−1850 nm, due to the presence of C–H molecular overtone transitions in these regions [[16], [17], [18]]. The absorption spectrum of lipids in both the 1100−1300 nm (second overtone of C–H bonds) and 1650−1850 nm (first overtone of C–H bonds), shows well-differentiated peaks with higher absorption coefficients compared to the other main constituents of biological tissue, such as water and haemoglobin [[19], [20], [21], [22], [23], [24], [25], [26], [27], [28]]. Notably, the stronger absorption of lipids in the first overtone region when compared to the second overtone region (∼6.3 times), recommend this band for discerning developmental changes in biological bodies non-invasively byways of intravascular photoacoustic imaging (IVPAI) [26]. PAM in the first overtone region has been reported on white matter in a rat spinal cord [20], intramuscular fat and Drosophila Melanogaster larva [28] using optical resolution (OR)-PAM mode, and on lipid-laden atherosclerotic plaques and human femoral arteries using IVPAI mode [21,[24], [25], [26]].

So far, in most of the reports mentioned above, nanosecond optical parametric oscillators (OPO) were employed as excitation sources for PA generation. Despite their broadband tunability with sufficiently high pulse energy density (PED) to excite a PA signal, OPOs present a high cost, a large footprint and low pulse repetition rate (PRR), making them non-ideal for compact and efficient PAM systems [28]. An alternative source that could circumvent the use of an OPO is based on supercontinuum (SC) generation [[28], [29], [30], [31], [32], [33], [34], [35], [36], [37], [38]], which can have spectral bandwidths spanning several octaves [28] and provide a brightness order of magnitude higher than a synchrotron [37]. The use of SC laser sources for the practice of MS—PA applications [39,40] and PAM based multimodal applications have been already reported [41,42]. However, the reported SC lasers are either limited by the emission wavelength or by the PED required for MS-PAM of lipids in the first overtone region of C–H bonds. Recently, an SC laser source pumped at 1047 nm, below the zero-dispersion wavelength (ZDW) of a photonic crystal fibre (PCF), has been used for lipid detection [28]. However, due to the limited PED in the first overtone region, multi-spectral photoacoustic microscopy (MS-PAM) could not be demonstrated in the first overtone region of C–H bonds. In a recent work, we presented a promising way of generating a suitable SC based on a commercial erbium-doped fibre amplifier and few meters of standard single-mode optical fibre (SMF-28), producing sufficient PED to perform *ex vivo* MS-PAM of lipids in the first overtone region [34].

In the current report, we demonstrate, for the first time to our knowledge, *in vivo* MS-PAM in the first C–H overtone region by further scaling up the PED of the SC laser. The high PED is achieved by increasing the output power of the pump and using a directly modulated diode (DMD) as the seed. The DMD based configuration was chosen to further exploit the flexibility in pulse duration and PRR. The SC source is based on a robust and compact design, using telecommunication range multiple-stage erbium (Er) and erbium: ytterbium (Er: Yb) co-doped fibre amplifiers and a few meters of standard dispersion-shifted fibre (DSF). When compared to the previously reported SC laser [34], the current source exhibits not only higher power spectral density (PSD) but also a significantly higher PRR, thus increasing the image acquisition speed.

In this paper, we further demonstrate the applicability of such a laser source by performing MS-PAM of lipids on *ex vivo* adipose tissue and *in vivo* on *Xenopus laevis* tadpoles over the entire first overtone region. The system can visualise lipid distribution inside both samples with high contrast, thus carving out a new direction towards compact, broadband and cost-effective sources for label-free imaging of lipids in both developmental biology and medical applications.

## 2 Materials and methods

### 2.1 Ethical approval

*In vivo* experiments were performed on the *Xenopus laevis* tadpole at developmental stage 37/38, based on Nieuwkoop and Faber 1956 [43]. Embryos were supplied by the European *Xenopus* Resource Centre (EXRC, Portsmouth UK) and kept at ∼20 °C in tap water. During the MS-PAM experiments, animals were anaesthetised in 0.1 % MS-222 solution (ethyl 3-aminobenzoate methanesulfonate, Sigma-Aldrich). All experimental procedures on stage 37/38 tadpoles are unregulated but were nevertheless approved by the University of Kent's animal welfare ethics committee.

### 2.2 MS-PAM system

**Fig. 1:**
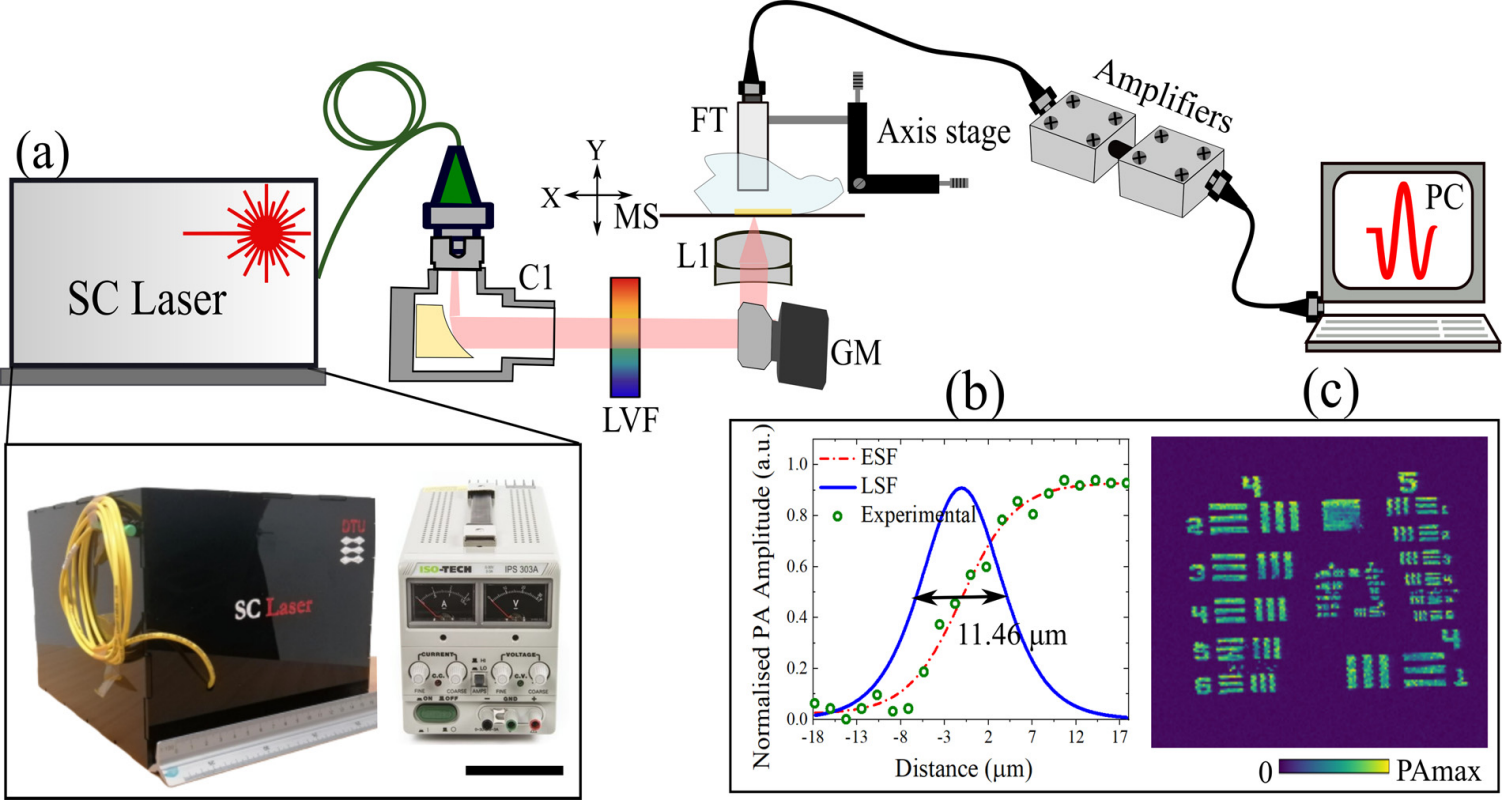
(a) Schematic of the MS-PAM system. C1: reflective collimator, LVF: linear variable filter, GM: galvo-mirrors, L1: achromatic lens, MS: microscopy slide, FT: flat transducer, PC: the personal computer. The photograph in the bottom left shows the all-fibre SC laser and the power supply unit with a scale bar of 10 cm. (b): Lateral resolution of the MS-PAM system estimated by using the edge and line spread functions. (c): PAM image of the USAF resolution target at 1720 nm.

Fig. 1(a) shows a schematic overview of the MS-PAM set up together with a photo of the actual all-fibre SC laser used in our experiments, including the power supply. The system is based on a custom in-house fabricated fibre-coupled SC laser as the excitation source. The laser beam from the SC is collimated using a broadband reflective collimator (RC04FC-P01, Thorlabs) and then spectrally filtered by a linear variable filter (LVF) (LVF 1.2–2.5-3.5-15-0.5, Vortex Optical Coatings). The filtered excitation beam is steered using a set of orthogonal galvanometer-based XY scanners (6220H, Cambridge Technology Ltd), allowing raster scanning of the sample in the lateral directions. A mounted achromatic lens doublet (AC254-040-C-ML, Thorlabs) was used to focus the excitation beam onto the sample. A custom-made ultrasonic transducer (COPAC) with a centre frequency of 10 MHz detects the photoacoustic (PA) signals generated by the optical excitation bands. The detected PA signal is amplified using two low-noise wideband amplifiers (ZFL-500LN, Mini-Circuits) and then conveyed towards the input of a fast digitizer (PCI-5124, National Instruments) for data processing. The digitizer, hosted in a PC, operates at 200 MS/second, allowing signal up to a maximum bandwidth of 100 MHz. An internal hardware filter of the digitizer was configured as a low pass filter up to 40 MHz. As the repetition rate of the source is 100 kHz, each temporal analogue signal of 10 μs is generated by the transducer. Each pulse is digitised into 2000 sampling points within an interval of 10 μs, while the optical beam is scanned over the sample. However, in order to improve the signal-to-noise ratio (SNR) and maintain an isometrically resolved image, the signal is cropped into 410 points around the area of interest. A triangular signal drives the fast galvo-scanner at 122 Hz, the ascending part of the triangular signal is used for the acquisition while the descending part of processing the data.

In this way, since the optical source was set at a repetition rate of 100 kHz, each generated B-scan image is assembled from 410 lateral pixels. As a consequence, a complete 3D volumetric image of size 410 × 410 × 410 pixels is acquired in 3.2 s.

After each lateral scan of the optical beam over the sample, the signal is processed by a Hilbert transform to obtain the envelope of the sample’s PA signal to deliver the PA depth profile (A-scan) for each lateral pixel and then assemble the depth profiles to obtain a B-scan. As the time required to process 410 A-scans was 8.2 ms (the time required by the fast galvo-scanner to return to its initial position), B-scan images were displayed in real-time at a frame rate of 122 Hz.

All the samples used in the experiments were placed in Petri dishes and immersed in either clear ultrasound gel for *ex vivo* experiments or in a water-based solution for *in vivo* experiments, to facilitate acoustic coupling. As the Petri dish available does not exhibit sufficient transparency in the spectral window of interest, an imaging window was created at the bottom of the dish and sealed with a microscope slide (thickness of 0.2 mm). Two independent 3D translation stages were used to support and coaxially align the sample holder and transducer to the optical excitation beam.

The lateral resolution of the MS-PAM system was experimentally quantified by imaging the edge of an element on a positive 1951 United States Air Force test target (R1DS1P, Thorlabs). As shown in Fig. 1(b), the edge spread function (ESF) was calculated by fitting the raw PA signal collected from scanning the edge in steps of 1.6 μm. The line spread function (LSF) was then calculated by taking the derivative of the ESF. The lateral resolution of the system is defined as the full width at half-maximum (FWHM) of the LSF and was found to be ∼11.46 μm. The theoretical lateral resolution of the MS-PAM system at the beam focus was calculated to be ∼10.79 μm at a 1720 nm excitation wavelength, indicating that the system is close to the diffraction limit. The photoacoustic maximum amplitude projection (MAP) of the test target is shown in Fig. 1(c).

## 3 Results and discussion

### 3.1 Optical characterisation of the SC laser

**Fig. 2:**
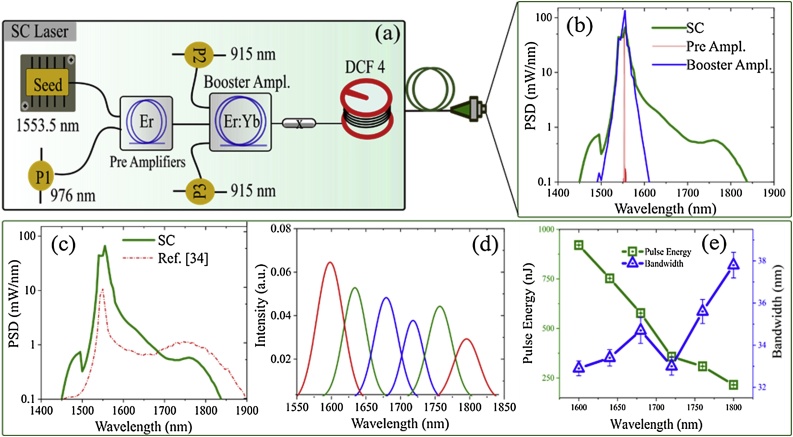
(a) Schematic of the SC laser configuration. DCF4: dispersion-shifted fibre. (b): PSD of preamplifier, booster amplifier and the SC spectrum generated by pumping a 4.5 m non-zero DSF. (c): SC output spectrum (solid line) in comparison with the SC reported in our previous work (dashed line). (d): SC Excitation bands filtered using the LVF. (e): Pulse energy and bandwidth of filtered excitation bands from the SC laser.

As shown in Fig. 2(a), the seed laser (DFBITU-100, EMCORE), providing an output power of 20 μW at 1553.5 nm, is directly modulated to produce 7 ns pulses (FWHM) at a PRF of 100 kHz. Two high-gain pre-amplifier stages amplify the pulses from the directly modulated seed laser, each comprised of a 3.5 m long Er-doped optical fibre (PM-ESF-7/125, Nufern) with a core diameter of 7 μm. This optical fibre was core pumped using a single 976 nm CW laser diode (IIVI-LD976-6, II—VI) in the forward configuration. The average output power measured after the first and second preamplifiers were ∼ 3 mW (17 dB optical gain) and 101 mW (15.2 dB optical gain) respectively. In the booster amplification stage, a 4.5 m co-doped Er: Yb fibre (PM-EYDF-12/130, Nufern) with a core diameter of 12 μm was cladding-pumped using two 915 nm CW multimode high-power laser diodes (MU20-915-01/02, Oclaro) in the backward configuration. A 2 nm bandpass filter after the preamplifier, is used to suppress amplified spontaneous emission (ASE) and parasitic lasing. The average output power measured after the booster amplifier is 2270 mW (with 9 W pump power, corresponding to a 25.2 % efficiency, which is equivalent to a pulse energy of 22.7 μJ per pulse at 100 kHz repetition rate.

The output from the booster amplifier is used to pump 4.5 m of a commercially available DSF (DCF 4, Thorlabs), thereby generating an SC from 1440−1870 nm, with an average output power of 1830 mW. SC generation is initiated by modulation instability (MI), which breaks up the long pump pulses into large numbers of solitons that undergo Raman self-frequency shifting and collisions, which further extend the spectrum towards longer wavelengths of up to 1870 nm [[29], [30], [31]]. To avoid back reflections from the end facet of the DSF, the output end of the DSF was angle cleaved and then connectorized (FC/APC-30126A9, Thorlabs) for ease of handling.

The PSD of the pre-amplifier (red), booster amplifier (blue) and the SC (green) are shown in Fig. 2(b). Fig. 2(c) shows the PSD of the SC from the current SC laser configuration (green) in comparison to our previous work (red). The higher PSD of the SC can be attributed to the higher pump power due to additional amplification stages. An LVF with a bandwidth of around 2 % of the centre wavelength (33−38 nm) is used to filter the excitation bands from the SC. Figs. 2(d) and 2(e) show the six filtered excitation bands from the SC used during the multi-spectral experiments and their respective pulse energies and the bandwidth in the individual bands. It can be noticed that all excitation bands have sufficiently high pulse energy (> 197 nJ) for performing OR-PAM [32].

### 3.2 *Ex vivo* MS-PAM imaging of Adipose tissue

**Fig. 3:**
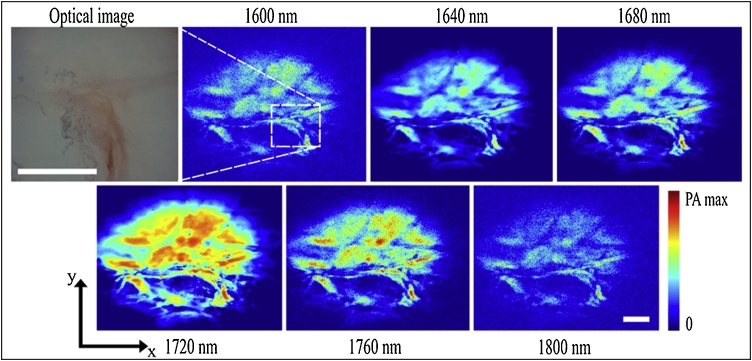
Optical image and MS-PAM images of *ex vivo* adipose tissue. Six z-projected *en-face* MS-PAM images are acquired from 1600 nm to 1800 nm in steps of 40 nm. The white bar in the optical image and the last MS-PAM image at 1800 nm represents the scale bar of 1 mm.

MS-PAM imaging was performed on a thin slice (about 3 mm) of adipose tissue from sheep in the 1600−1800 nm wavelength range. Six excitation bands in steps of 40 nm were filtered from the SC laser using the LVF, shown in Fig. 2. The acquisition and analysis of the raw data are accomplished by using in-house software developed in LabVIEW. The programme instructs a fast digitizer to convert the analogue to digital signal collected by the transducer in synchronism with the optical pulses generated by the source and the start and stop of lateral scanning by the galvo-scanners. For each position of the beam on the sample, the PA signal is digitised, and an A-scan is generated using a Hilbert transform. An intensity graph is produced by plotting the PA amplitudes extracted from each A-scan recorded as a function of X and Y position to form a PAM image, an. As mentioned earlier, a complete 3D volumetric image of size 410 × 410 × 410 pixels is acquired in 3.2 s. To improve the SNR; however, as conventionally done in PAM, data acquisition is repeated for 16 times, and signals averaged (averaging over 16 A-scans) before generating a B-scan. Therefore, the PAM images demonstrated in Fig. 3, require 51.2 s. This technique enhances the contrast in the image.

Fig. 3 shows an optical image and MS-PAM images of *ex viv*o adipose tissue that are z-projected *en-face* images at six different excitation bands. The images are normalised to their respective pulse energy in the excitation band used.

**Fig. 4:**
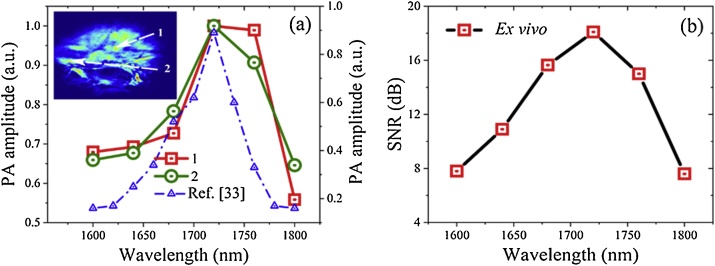
(a) Normalized PA amplitudes at two different regions (labelled 1 and 2 in the inset) for the six excitation bands with corresponding PA amplitudes for adipose tissue measurements reported in our previous study [33]. The area of each region is 0.087 mm^2^. (b) Measured spectral variation of the SNR of ex *vivo* adipose tissue measured in MS-PAM images.

The *ex vivo* adipose tissue images reveal a peak in the PA amplitude at 1720 nm, which is consistent with the PA spectrum measurements reported in our previous report [34]. PA spectra of the tissue acquired from two different regions are plotted in Fig. 4(a), both regions confirmed a stronger PA absorption at 1720 nm, which can be attributed to the stronger absorption of lipids at 1720 nm due to the first overtone transition of C–H bonds, [21,25,28,34]. The SNR (ratio of the measured PA signal to the overall noise at each A-scan) of MS-PAM images at all six excitation bands is calculated and plotted in Fig. 4(b). As expected, the SNR is higher at 1720 nm (18.1 dB) when compared to other excitation bands as the lipid absorption is higher at 1720 nm.

### 3.3 *In vivo* MS-PAM imaging of the *Xenopus laevis* tadpole

**Fig. 5:**
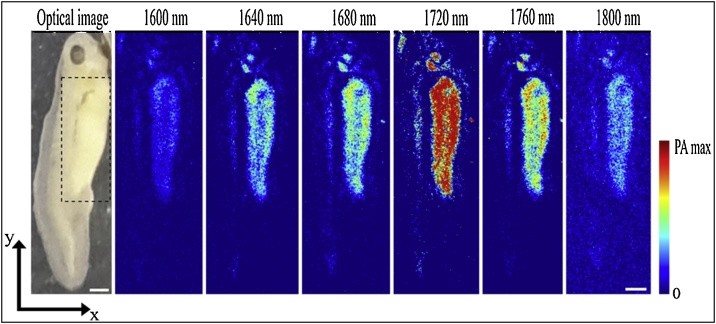
Optical image and 6 *in vivo* z-projected *en-face* MS-PAM images of a *Xenopus laevis* tadpole acquired from 1600 nm to 1800 nm in steps of 40 nm. The highlighted region in the optical image shows the yolk sac. The scale bar represents 1 mm.

*Xenopus laevis* tadpoles were raised in water to stage 37–38, about 53 h post-fertilisation. Tadpoles were chosen at this early stage of development for MS-PAM imaging because they allow easy access to the yolk sac, a well-defined area (Fig. 5) with a high concentration of lipids [44]. Moreover, the skin covering the yolk sac lacks pigmentation, which permits unobstructed laser penetration in the intact animal. Laser radiation in the excitation bands throughout the experiment is well within the maximum permissible exposure (MPE) level. The MPE for tissue is 1 J/cm^2^ for ns pulses and for the SC excitation band with maximum PED (914 nJ in the 1600 nm excitation band), the exposure level is about 0.88 J/cm^2^.

Fig. 5 shows a photo of the tadpole together with z-projected *en-face* MS-PAM images of the whole body at the six different excitation bands. The MS-PAM images are averaged over 16 A-scans and normalised to the respective pulse energies in the excitation bands. Again, a strong signal is observed at 1720 nm excitation due to the high concentration of lipids, and the contrast decreases at all other excitation wavelengths. As it can be seen from the MS-PAM images of the tadpole, the yolk sack section of the tadpole shows PA signals, which are distributed throughout the section all along its anterior-posterior and the ventral-dorsal axis. The strong PA signals from the yolk sac can be attributed to the presence of vast reserves of lipid molecules [44], which have strong optical absorption due to the first overtone transition of the C–H bond. This agrees with the known abundance of the lipoprotein Vitellogenin in the Xenopus laevis yolk sac [44].

**Fig. 6:**
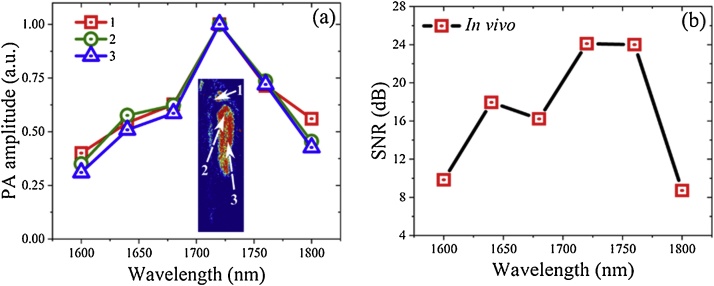
(a) Normalized PA amplitudes of a small region (about 0.087 mm^2^) in the MS-PAM images of a tadpole at three different places (labelled 1, 2, and 3) for the six excitation bands. (b) Measured SNR of tadpole MS-PAM images at all the six excitation bands.

Fig. 6(a) shows the PA spectra of three different regions (Size of 0.087 mm^2^ each) in the tadpole MS-PAM images measured at the 6 excitation bands as above. The PA signals demonstrate consistent results showing the expected maximum at 1720 nm. The SNR of the *in vivo* images at the six excitation bands is calculated and displayed in Fig. 6(b).

Again, the SNR is higher at 1720 nm when compared to other excitation bands. SNR for *in vivo* MS-PAM images are higher when compared to the SNR for *ex vivo* MS-PAM images as the image scan area used for *in vivo* experiments is smaller than in case of the *ex vivo* experiments.

## 4 Conclusions

A high-pulse energy all-fibre SC source based on standard telecom range optical components for MS-PAM imaging of lipids is demonstrated. The filtered optical pulses from the SC source have sufficient pulse energy density for MS-PAM studies on lipids over the first overtone transition of C–H vibration bonds (1650–1850). By employing the SC laser in conjunction with an LVF, we demonstrated the applicability of such a laser source by performing MS-PAM imaging of lipids in *ex vivo* adipose tissue and *in vivo* using *Xenopus laevis* tadpole. Our study has shown that the MS-PAM system can visualise lipids in both *ex vivo* and *in vivo* tissues, which makes it applicable for a wide range of applications. We believe the proposed high pulse energy SC laser paves a new direction towards more compact sources for label-free imaging of lipids in both developmental biology and medical imaging. In future, we would like to scale the PED of the SC laser by using large mode area fibres in the MOPA configuration so as to perform the MS-PAM with higher SNR. Moreover, the higher PED of the laser will allow the use of narrow bandpass filters (like AOTFs) which thereby enable high-resolution MS-PAM of multiple endogenous biological molecules (lipids, glucose, water and collagen) which reveal dominant absorption features in the emission wavelength (1540−1840 nm) of the developed source.

## Declaration of Competing Interest

The authors declare that there are no conflicts of interest
